# Health Behaviour among Nurses Working in Public Hospitals in Kakamega County, Kenya

**DOI:** 10.1155/2017/4683189

**Published:** 2017-12-31

**Authors:** Mchidi Kiguhe Nebert, B. M. Okello Agina, Yitambe Andre

**Affiliations:** ^1^Department of Community Health, School of Public Health, Kenyatta University, GPO 43844-00100, Nairobi, Kenya; ^2^School of Medicine, Kenyatta University, GPO 43844-00100, Nairobi, Kenya; ^3^Department of Health Management and Informatics, School of Public Health, Kenyatta University, GPO 43844-00100, Nairobi, Kenya

## Abstract

Health behaviour refers to actions undertaken by a person who perceives self to be ill for the purpose of finding an appropriate remedy. Nurses as gate keepers of health are expected to seek formal treatment when they are taken ill because this is what they teach their patients. Nurses' working conditions all over the world are described as squalid with long working hours and workload. This scenario predisposes them to occupational health hazards and at the same time denies them time for self-care. Although nurses are knowledgeable about disease and its treatment and have access to health care, they engage in self-treatment in contrast to what they teach patients. Health behaviour among nurses in Kakamega County was investigated using a cross-sectional design. Data was collected using self-administered questionnaires and subjected to bivariate and logistic regression analyses. The study found that health behaviour of nurses in Kakamega County is below expectation, as 33% (*n* = 61) engaged in voluntary screening services. Further, 34.8% (*n* = 65) said that their health would improve if they engaged in health promotion activities. The study recommends empowering nurses to engage in positive health behaviour through education. The county should also provide affordable screening services to its nurses.

## 1. Background

Nurses are an important resource for health. They have been cited as the backbone of health care provision due to their numerical strength in any health setting and being the most trusted profession among patients makes them ideal patient educators [1, 2]. In order to be realistic in their health education and patient expectations, nurses must lead from the front by doing as they expect of their patients by engaging in positive health behaviour [3]. Health behaviour refers to actions of individuals aimed at detecting or preventing disease and improving their well-being [4]. Due to endemic shortages of health care providers all over the world, nurses are and will continue to be exposed to risks that predispose them to poor health as a result of increased workloads and long working hours that lead to burnout [5–8]. These situations limit their ability to achieve a work-life balance [9, 10]. Nurses lack self-care discipline and engage in poor feeding habits and are burdened with noncommunicable diseases, circumstances that embarrass their statures as role models to their patients [11]. Nurses, like doctors, think that they are omnipotent and invisible, but there is increased morbidity among them due to the demanding nature of their work [12–17].

An Israeli study found that although doctors had strong belief in screening tests, only 27.5% of the respondents had undergone the tests, with 55.6% blaming it on lack of time [18]. Further, a Baltimore study found more than half of the participants as not having a regular meal schedule, leading to poor eating habits and obesity [19]. A Jordanian study found cultural beliefs as significant in determining the practice of self-breast exam for cancer screening among female nurses. A Norwegian study found that physicians were reluctant to attend screening programs due to forgetfulness or lack of time or due to the belief that they knew their own health to the extent that they thought they were at low risk for disease [5], factors that are cross-cutting in medical profession. Medicine has the professional culture of discomfort in seeking help [20, 21]. Evidence shows that consultations that do not follow laid down protocols are rampant within the medical profession [22]. This unfortunate scenario has been attributed to perceived trust one has in the physician they choose for fear of confidentiality breach if they follow laid down protocols [16, 23]. Confidentiality dilemma reinforces what Wallace et al. [20] call “a conspiracy of silence” within the profession and contributes to negative health behaviour. Because health providers determine the confidentiality of health information they receive regarding patients, lack of trust in the confidentiality of the information nurses share with their peers regarding their health may be a barrier to health care seeking [24]. In Kenya, Taegtmeyer et al. [25] found that health care providers who had had a needle stick injury did not seek treatment because of the fear of HIV testing.

## 2. Problem Statement

In Kakamega County, anecdotal evidence suggests that the work load is very high, with many nurses reporting to work while sick. For example, the paediatric ward with a bed capacity of 34 usually has two nurses working on a shift. The county is the second populous county in Kenya, yet its nurse patient ratio of 34.87 : 100,000 is below the national average of 51.5 : 100,000. The most populous county, Nairobi, with double the population, has a nurse patient ratio of 88.74 : 100,000 [26]. This means that the nurse in Kakamega County has a lot of work pressure to meet patient health care needs, a situation that could compromise not only the nurses' health but also the health of the patients they take care of. From this background, there is need to investigate health behaviour of the nurses and generate information about them, an area that has not been studied in Kenya.

## 3. Specific Objectives

The objectives of this paper are listed as follows:

1. To identify the predisposing factors influencing health behaviour of nurses in Kakamega County
2. To determine the enabling factors influencing health behaviour of nurses in Kakamega County
3. To investigate the need factors that shape health behaviour of nurses in Kakamega County

## 4. Materials and Methods

The study was conducted at the Kakamega County in Western Kenya. Kakamega County has 1 county referral hospital, 4 county hospitals, 7 subcounty hospitals, 34 health centers, and 86 dispensaries run by the government and several health facilities run by faith-based organizations and nongovernmental organizations. Within the county, nurses working in select public health facilities were selected. Specifically, the health facilities were 4 subcounty hospitals, 2 county hospitals, and 1 county referral hospital as listed in Table 1. The sample size was 187 nurses, and respondents were selected using simple random sampling technique. Data was collected using self-administered questionnaires using adaptations from a questionnaire used by Chen et al. [27]. Need factors were assessed using modifications of the SF-12v2 [28]. Ethical clearance was obtained from Kenyatta University Ethics Review Committee and a permit to carry out the study was obtained from the National Council for Science, Technology and Innovation.

Univariate analysis was used to describe the distribution of each of the variables. Bivariate analysis was used to investigate the difference between health behaviour and the predisposing, enabling, and need factors. Level of significance was set at *p* ≤ 0.05 (95% confidence interval).

## 5. Results and Discussion

### 5.1. Predisposing Factors Influencing Health Behaviour

The majority belonged to age bracket of 30–39 years; the modal age was 40 years, while the median and mean age was 41 years, respectively. As shown in Table 2, majority of the respondents were female Protestants and had Kenya Registered Community Health Nurses (KRCHN) diploma as highest level of nursing qualification. The majority of the respondents were married, reported moderate support from supervisors, and had worked as nurses for more than 10 years.

### 5.2. Enabling Factors Influencing Health Behaviour

As shown in Table 3, the majority of the nurses said that work load was very high and they worked for 40–50 hours a week. Although the majority of the respondents said that they would prefer to be treated in the facility they worked in, they expressed dissatisfaction with both the quality of and access to health services available to them in the county. The most common health insurance cover possessed by the respondents was the National Health Insurance Fund (NHIF); the majority of respondents felt that this insurance cover was inadequate in meeting their health expenditure.

### 5.3. Need Factors Influencing Health Behaviour

As Table 4 shows, the majority, 44.9% (*n* = 84), rated their current health at the time of study as good and as being about the same state as compared to a year prior to the study. Regarding how nurses rated their physical and psychological health in comparison with that of other individuals of the same age and gender, the majority rated it as better. 34.2% of the nurses were extremely concerned about their health within 12 months preceding the study and were optimistic that their health would be good in 2 years from the time of study.

**Figure 1:**
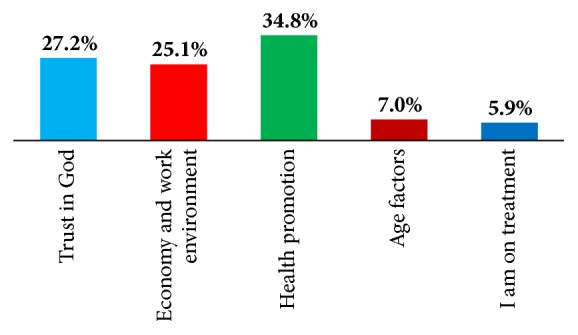
Factors that will influence nurses' health outlook in 2 years.

Asked for the reason as to why they said their health would be as forecasted, Figure 1 shows the reasons for the projection. Those who did not have positive prospects about the future outlook of their health cited age-related factors and poor economic returns for the future as barriers to achieving their ideal health status.

### 5.4. Health Behaviour of the Nurses

Health behaviour investigated self-reported voluntary actions of the respondents aimed at detecting or preventing disease and improving well-being. This outcome variable investigated if nurses engaged in voluntary screening services for the purpose of detecting disease.

### 5.5. Voluntary Screening Undertaken by the Nurses

**Figure 2:**
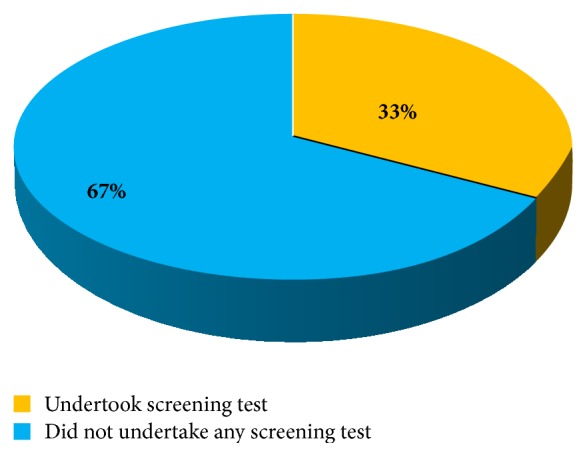
Uptake of voluntary screening services by nurses.

With regard to voluntary screening services independent of a request from a health provider in the last 12 months, Figure 2 shows that 67% had never undertaken any voluntary screening service in the year preceding the study. Of these, the majority, 65.6%  (*n* = 40), were females, while 34.5%  (*n* = 21) were males.

### 5.6. Bivariate Analysis

The chi-square test was used to show if there existed significant differences between predisposing, need, and enabling factors to health behaviour as shown in Table 5. The only variable that was statistically significant was the nurses' concerns about the future prospects. The finding shows, though without significance difference, that being female, increasing age, being married, being a Protestant, having diploma qualification, having longer working experience, good supervisor support, and being worried about future health positively influence health behaviour.

### 5.7. Types of Voluntary Screening Services Undertaken by Nurses

The most prevalent voluntary screening services reported to have been undertaken included HIV screening, cervical cancer screening, breast cancer screening, diabetes screening, and screening for hypertension. All the respondents reported to have undertaken multiple tests.

## 6. Reasons for Undertaking Voluntary Screening Services by the Nurses

**Figure 3:**
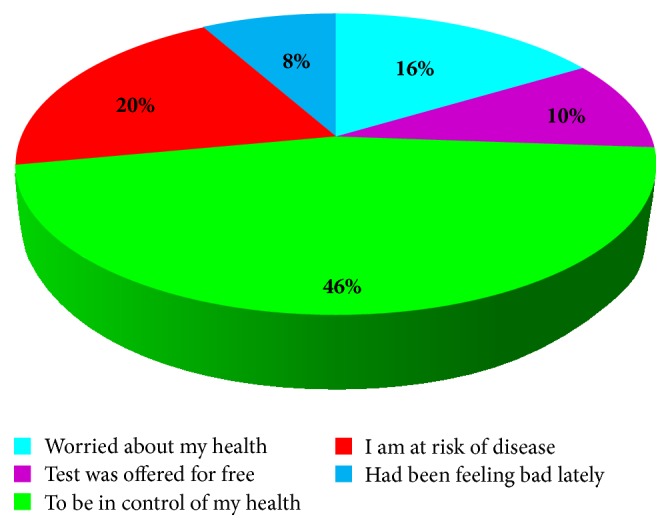
Reasons for undertaking screening tests by the nurses.

Figure 3 shows that, for those who undertook voluntary screening services (*n* = 61), the majority (46.0%) said they wanted to be in control of their health, 20.0% said they knew they were at risk of disease, 16.0% said they were worried about their health, and 10.0% said the test was offered for free, while 8.0% said they were concerned about their health.

## 7. Barriers to Undertaking Voluntary Screening Services by the Nurses

**Figure 4:**
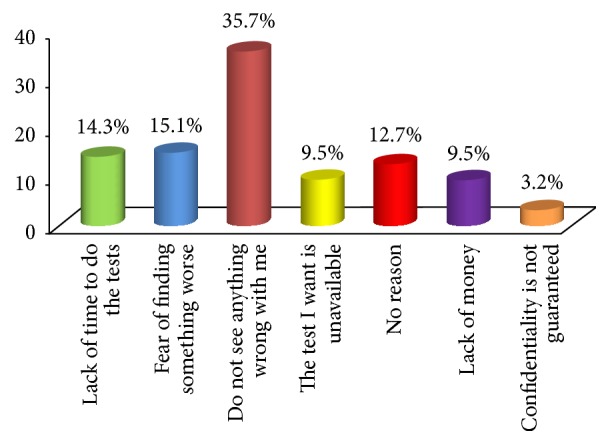
Reasons for not undertaking screening tests by the nurses.

Figure 4 shows that, for those who had never undertaken any voluntary screening tests in the said period (*n* = 126), the majority (35.7%) said they saw nothing wrong with themselves, 15.1% said they feared finding something worse, 14.3% said they lacked time to do the tests, 12.7% said they did not have a reason, and 19% said lack of both money and the test they wanted prevented them, while 3.2% attributed it to fear for their confidentiality not being guaranteed.

### 7.1. Chronic Illnesses Reported by the Nurses

The majority, 70.6%  (*n* = 132), of the nurses reported that they did not suffer from any chronic illness. This was followed by 27.3%  (*n* = 51) who said they suffered from at least one chronic illness and lastly 2.1%  (*n* = 4) reported that they did not know if they suffered from any chronic illness. The prevalence of hypertension was highest at 9.1%  (*n* = 17), followed by diabetes mellitus at 2.7%  (*n* = 5) and HIV at 1.1%  (*n* = 2) among others.

## 8. Discussion

With regard to age, the majority of the nurses belonged to age bracket of 30–39 years and their mean age was 41 years. This finding is consistent with Ministry of Health's [26] report that showed that the majority of Kenyan nurses were in age bracket of 31–40 years. Similarly, Wakaba et al. [29], while investigating the public sector nursing workforce in Kenya, found the mean age of nurses to be 44 years.

The respondents who rate their health as good reported utilizing voluntary services more; this is a paradox because it is not clearly evident on the basis of this study to establish whether the good rating of health was a result of a positive screening result or whether the self-appraisal of poor health was a barrier to screening services uptake with fear of finding something worse as a real fear. Fear of finding something worse as a barrier to screening tests is well documented in literature; for example, Frank and Segura [5] found that health professionals did not undertake screening tests because of fear, while Lindo et al. [30] found the barrier to be related to confidentiality within the health profession. Another barrier to screening tests was the fact that nurses saw nothing wrong with their health. This report was common in the young, those with a higher diploma and above, and those who had worked for less than five years in the county. Despite individual differences, one expects that a profession that is the gate keeper of health should be in the frontline not only advocating for but also undertaking screening tests knowing that they are at risk of disease, but that is contrary. This can be attributed to the fact that nurses feel invincible and believe that illness belongs to patients. Although nursing is a calling to serve humanity, not actively taking the lead in engaging in health preventive behaviour and leaving the future health prospect to* “the will of God”* is in itself a barrier that nurses in Kakamega County must overcome in the pathway to personal health locus of control because the realisation of vision 2030 is hinged on health [31], which must first start with that of the caregivers, the nurses.

Regarding the prevalence of life-limiting illness, the majority reported suffering from hypertension, followed by those who suffer from diabetes, with 0.5% of the respondents volunteering their HIV-positive status. It is worthwhile to note that whereas the majority of the nurses had never undertaken a screening test, they reported having no chronic disease, an interesting finding that could corroborate invincibility. The finding is consistent with that of Canbulat and Uzun [32] who found that the screening practices of health workers were low, which contributed to the perceived low levels of life-limiting illnesses in health workers. The low level of screening tests undertaken accrues from the fact that the majority of the respondents see nothing wrong with themselves and therefore have not undertaken screening tests, yet it is known that life-limiting illnesses in their infancy are asymptomatic. Peleg et al. [18] found that doctors had strong beliefs in screening tests but these beliefs did not translate to personal uptake. The fear, invincibility, and the belief that the nurses are the barometer for public's health status are misleading and must be addressed if nurses want to be better role models for the public.

## 9. Conclusions and Recommendations

The study revealed high proportion of nurses that did not undertake screening services voluntarily. As nurses, it is expected that they not only teach the public about the benefits of health behaviour but also lead as example in undertaking the voluntary screening tests to detect early disease. This study has uncovered the existing barriers to uptake of screening tests among nurses. These barriers are crosscutting in the biosocioeconomic sphere of the nurses. Further, cost is an important barrier to uptake of screening services coupled with the finding that Kakamega County is not well endowed with the resources that are necessary for voluntary screening services among nurses. This study recommends that there is a need to promote health behaviour of the young and male nurses to uptake screening services more; this may mean more education. Nurses also need to be sensitized to undertake screening tests for both communicable and noncommunicable diseases not only for the purpose of leading from the front but also so that the Kakamega County population is served by a healthy work force, from which they draw inspiration. It is also important that the prioritization of scarce resources for health care in Kakamega County be addressed so that adequate resources are allocated for screening services to enable nurses to undertake screening services more.

## Figures and Tables

**Table 1:** Distribution of nurses in the selected facilities in Kakamega County (source, County Chief Officer of Health, 2013).

District	Name of facility	Number of nurses
Kakamega central	Kakamega County Referral Hospital	241
Malava	Malava Sub-County Hospital	31
Butere/Mumias	Butere Sub-County Hospital	31
	Matungu Sub-County Hospital	25
Lugari	Lumakanda County Hospital	25
Likuyani	Likuyani County Hospital	17
Ikolomani	Iguhu Sub-County Hospital	16
*Total*		*386*

**Table 2:** Univariate analysis of predisposing factors that influence health behaviour.

Characteristics	Frequency (*n*)	Percent
Age (years)		
Below 30	24	12.8
30–39	55	29.4
40–49	54	28.9
50 and above	54	28.9
*Total*	*187*	*100.0*
Gender		
Male	64	34.2
Female	123	65.8
*Total*	*187*	*100.0*
Marital status		
Married	143	76.5
Single	30	16.0
Separated/widowed	14	8.5
*Total*	*187*	*100.0*
Religion		
Protestant	147	78.6
Muslim	2	1.1
Catholic	38	20.3
*Total*	*187*	*100.0*
Highest level of nursing qualification		
Postgraduate level	5	2.7
BScN	26	13.9
Higher diploma in nursing	13	7.0
KRCHN (diploma in Nursing)	105	56.1
ECN (certificate in Nursing)	32	17.1
KRN/M (registered midwife)	6	3.2
*Total*	*187*	*100.0*
Years nurses have worked in the county		
6 months–1 year	21	11.2
1 year–5 years	41	22
6 years–10 years	35	18.7
More than 10 years	90	48.1
*Total*	*187*	*100.0*
Support from immediate supervisor		
Very low	18	9.6
Low	17	9.1
Moderate	65	34.8
High	63	33.7
Very high	24	12.8
*Total*	*187*	*100.0*

**Table 3:** Univariate analysis of enabling factors that influence health behaviour.

Characteristics	Frequency (*n*)	Percent
Preferred treatment source		
Public facility	97	51.9
Private facility	85	45.4
Self-treatment	5	2.7
* Total*	*187*	*100.0*
Insurance cover possessed by nurses		
NHIF	180	96.3
UAP	2	1.1
Jubilee	4	2.1
CIC	1	.5
* Total*	*187*	*100.0*
Satisfaction with the adequacy of insurance cover		
No	103	55.1
Yes	84	44.9
* Total*	*187*	*100.0*
Number of hours that nurses in Kakamega County spend working per week		
Less than 40 hours	7	3.7
40–50 hours	124	66.3
More than 50 hours	56	30
* Total*	*187*	*100.0*
Work load of nurses in Kakamega County		
Low	1	0.5
Moderate	17	9.1
High	52	27.8
Very high	117	62.6
* Total*	*187*	*100.0*
Satisfaction with the health services that you have access to in this county		
No	153	83.1
Yes	31	16.9
* Total*	*184*	*100*
Satisfied with the quality of health services that are available to you		
No	145	78.8
Yes	39	21.2
* Total*	*184*	*100.0*

**Table 4:** Univariate analysis of need factors that influence health behaviour.

Characteristics	Frequency (*n*)	Percent
Rating current health		
Poor	4	2.1
Fair	58	31.0
Good	84	44.9
Very good	25	13.4
Excellent	16	8.6
* Total*	*187*	*100.0*
Rating of nurses' general heath in comparison to last year		
Worse	8	4.3
Fairer	34	18.2
About the same	59	31.6
Good	58	31.0
Very good	28	15.0
* Total*	*187*	*100.0*
Rating nurses' comparison of their physical health with that of other individuals of the same age and gender		
Much worse than theirs	5	2.7
Somewhat worse than theirs	18	9.6
About the same as theirs	65	34.8
Better than theirs	70	37.4
Much better than theirs	29	15.5
* Total*	*187*	*100.0*
Rating nurses' comparison of their psychological health with that of other individuals of the same age and gender		
Much worse than theirs	7	3.7
Somewhat worse than theirs	25	13.4
About the same as theirs	53	28.3
Better than theirs	72	38.5
Much better than theirs	30	16.0
* Total*	*187*	*100.0*
Rating nurses health concerns about their health in the preceding 12 months.		
Not concerned at all	23	12.3
Slightly concerned	24	12.8
Somewhat concerned	29	15.5
Moderately concerned	47	25.1
Extremely concerned	64	34.2
* Total*	*187*	*100.0*
Rating how nurses projected their health in 2 years after the study		
Worse	14	7.5
Fairer	19	10.2
About the same	29	15.5
Good	65	34.8
Very good	60	32.1
* Total*	*187*	*100.0*

**Table 5:** Cross-tabulation of predisposing, enabling, and need factors and health behaviour.

Factors	Health behaviour (voluntary screening uptake)					
	Yes		No		*n*	*p* = ≤0.05
Gender						
Male	21	34.4	43	34.1	64	*χ* ^2^ = 0.002, df = 1, *p* = 0.968
Female	40	65.6	83	65.9	123	
* n*	*61*	*100*	*126*	*100*	*187*	
Age						
<30 years	9	14.8	15	11.9	24	*χ* ^2^ = 2.113, df = 3, *p* = 0.549
30–39 years	16	26.2	39	31.0	55	
40–49 years	21	34.4	33	26.2	54	
>50 years	15	24.6	39	31.0	54	
* n*	*61*	*100*	*126*	*100*	*187*	
Marital status						
Married	42	68.9	101	80.2	143	*χ* ^2^ = 2.920, df = 1, *p* = 0.087
Otherwise	19	31.1	25	19.8	44	
* n*	*61*	*100*	*126*	*100*	*187*	
Religion						
Protestant	44	79.3	103	77.5	147	*χ* ^2^ = 2.920, df = 1, *p* = 0.133
Catholic/muslim	17	20.7	23	22.5	40	
* n*	*61*	*100*	*126*	*100*	*187*	
Highest level of training as a nurse						
BScN/higher dip./postgraduate	17	27.9	27	21.4	44	
Diploma and below	44	72.1	99	78.6	143	*χ* ^2^ = 0.947, df = 1, *p* = 0.330
* n*	*61*	*100*	*126*	*100*	*187*	
Time working as a nurse						
6 months–5 years	23	37.7	39	31.0	62	*χ* ^2^ = 0.846, df = 1, *p* = 0.358
Above 5 years	38	62.3	87	69.0	125	
* n*	*61*	*100*	*126*	*100*	*187*	
Support from supervisor						
Low	27	44.3	64	50.8	91	*χ* ^2^ = 0.702 df = 1, *p* = 0.402
High	34	55.7	62	49.2	96	
* n*	*61*	*100*	*126*	*100*	*187*	
Worried about future health						
Worried	42	68.9	66	52.4	108	*χ* ^2^ = 4.570 df = 1, *p = 0.033*
Not worried	19	31.1	60	47.6	79	
*n*	*61*	*100*	*126*	*100*	*187*	

## Additional Points

*Limitations of the Study.* This study was cross-sectional in nature and therefore could not infer causation for any of the factors to health behaviour. The study relied on self-report, which has its inherent weakness.

*Areas of Further Research*. (1) Investigate health behaviour of all nurses using a nationwide sample so that there is a national reference point. (2) Investigate, through qualitative designs, the decision-making processes underlying health behaviour and barriers to health behaviour experienced by the nurses so as to uncover factors that enhance use of the reported health behaviour. (3) Investigate the determinants of access to quality health services to the nurses which can promote screening services uptake among nurses.
